# Blood Biochemical Biomarkers in Fish Toxicology—A Review

**DOI:** 10.3390/ani15070965

**Published:** 2025-03-27

**Authors:** Bartosz Bojarski, Małgorzata Witeska, Elżbieta Kondera

**Affiliations:** 1 Department of Animal Biology and Environment, Faculty of Animal Breeding and Biology, Bydgoszcz University of Science and Technology, Mazowiecka 28, 85-084 Bydgoszcz, Poland; 2 Department of Animal Environment Biology, Institute of Animal Science, Warsaw University of Life Sciences, Ciszewskiego 8, 02-786 Warsaw, Poland; malgorzata_witeska@sggw.edu.pl; 3 Institute of Biological Sciences, Faculty of Exact and Natural Sciences, University of Siedlce, Prusa 14, 08-110 Siedlce, Poland; elzbieta.kondera@uws.edu.pl

**Keywords:** fish, blood parameters, biochemical parameters, biomarkers, toxicity

## Abstract

Blood-based biochemical assays are used as predictive and diagnostic methods to evaluate fish welfare in aquaculture and research. The variations of blood biochemical parameters in fish are commonly used as biomarkers of exposure to toxic agents. Blood biochemical parameters can help identify the magnitude of toxicity and the mechanisms by which particular toxic agents act on the organisms. Some parameters typically measured in the blood can also be evaluated in the whole body in the early developmental stages of fish (embryos and larvae) that are often used in toxicological studies. This review assessed the usefulness of various blood biochemical indices as toxicity biomarkers. Analysis of multiple studies showed that toxicity-induced changes in most blood biochemical parameters in fish often depend on toxic agent concentration and exposure duration. Also, various parameters manifest different sensitivity to intoxication, and diverse directions of changes may occur. Among biochemical parameters, some are biomarkers of general physiological stress, while others indicate dysfunctions of particular organs. Moreover, hormonal endpoints seem to be sensitive but nonspecific biomarkers of intoxication in fish.

## Simple Summary

Analysis of blood biochemical parameters is used, among others, in the assessment of exposure to toxic chemicals. In this review, we show which biochemical parameters are frequently and rarely tested, provide examples of interpretations of test results, and discuss the usefulness of individual blood biochemical parameters in fish toxicology.

## 1. The Effects of Toxic Agents on Fish Metabolic Biomarkers

**Scheme 1:**
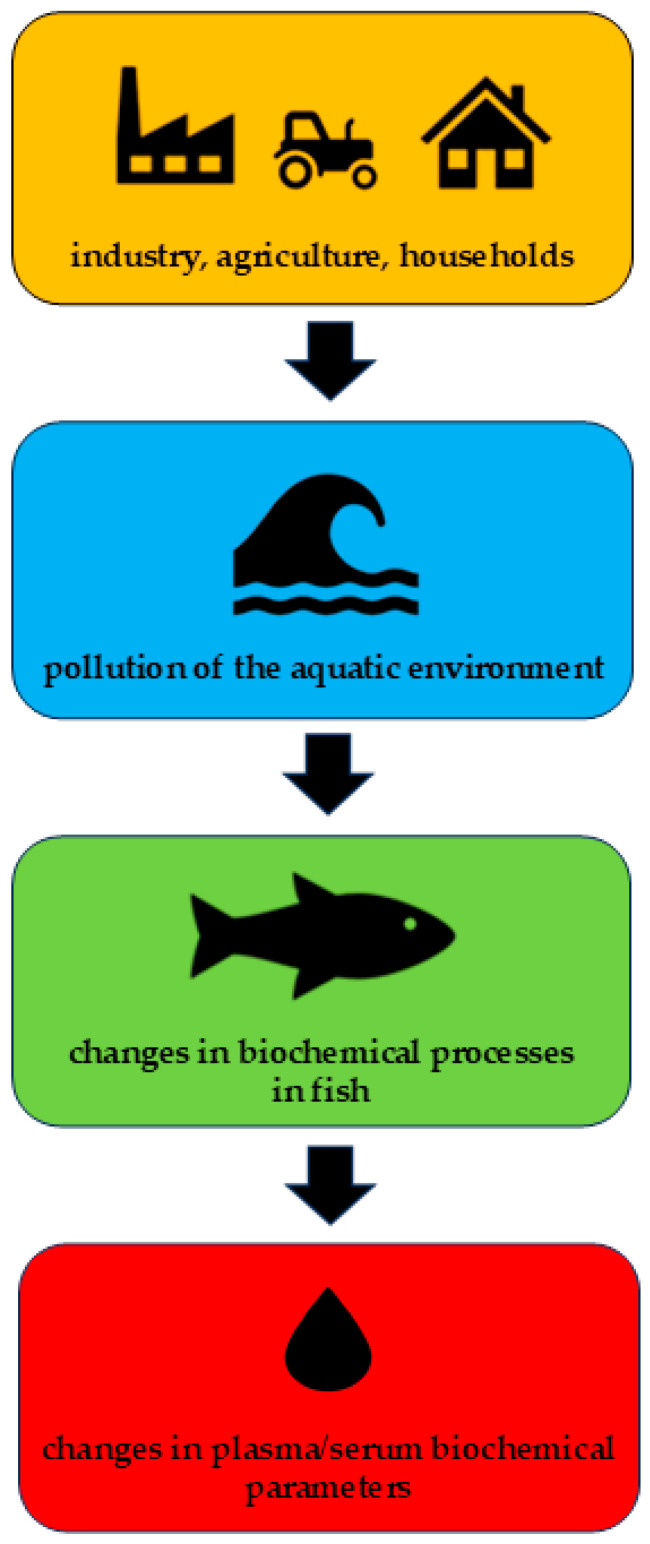
Changes in blood biochemical parameters in fish as a result of environmental pollution.

Due to pollution of the aquatic environment, fish are in constant contact with toxic substances, which leads to metabolic disorders that may manifest in changes in various blood biochemical parameters (Scheme 1). Metabolic parameters are widely applied biomarkers in assessing stress, welfare, and health conditions in fish. For example, the glucose concentration is an often used, easy, and cheap to measure blood biochemical parameter. Our analysis of results obtained by various authors showed that the blood glucose level was measured in 88% of the examples listed in Table 1. Increases in glucose concentration (39%) were observed about three times more often than decreases (14%), which suggests that hyperglycemia is a typical symptom of chemical stress in fish. However, no significant change was the most common observation (47%). A marked increase in glucose concentration in circulating blood is a part of the secondary stress response in fish and takes place in reaction to most types of stressors [1]. Hypoglycemia was typical for nanoparticle exposure and often occurred in fish exposed to insecticides (Table 1). It may result from increased energy consumption for detoxification processes or disturbed glycogenolysis or gluconeogenesis due to hepatic dysfunction. Moreover, our experience indicates that poisoning can cause cessation of feeding, which also leads to decreased blood glucose levels and exhaustion. The analysis of the data summarized in Table 1 showed that glucose levels were often unchanged or changed significantly depending on concentration and exposure duration. Sometimes, a statistically significant increase was first observed, and after a longer exposure time, there were no significant differences, which indicates adaptation to unfavorable environmental conditions. For example, exposure of *Oreochromis niloticus* to heavy metals resulted in increased glucose level after 4 days of exposure, while after 21 days of treatment, no significant changes occurred [2]. This result suggests that glucose is a rapidly responding marker of the early stage of intoxication. On the other hand, the blood glucose of *Cyprinus carpio* subjected to an organophosphate pesticide exposure at a lower concentration was unchanged after 10 and 20 days and increased after 30 days of treatment, while the higher tested concentration caused a significant increase in this parameter at each sampling point (10, 20, and 30 days) [3]. This suggests that glycemia depends on the level of exposure and resulting physiological stress, which confirms the limited sensitivity of this biomarker.

Total protein measurement is a laboratory test to assess all proteins’ concentration in the circulating blood plasma. Laboratory analysis of the total protein level in the blood helps diagnose various liver, kidney, and intestinal disorders. It is also a parameter indicating the nutritional status of fish [4,5,6]. The analysis of the collected data showed that hypoproteinemia (39%) was almost four times more common than an increase in total protein concentration (11%). However, it was impossible to indicate a group of toxic substances for which an increase or decrease in this parameter is typical. Moreover, in many cases, TP concentration was unchanged (50%). Nevertheless, TP level is often measured in toxicological experiments, especially with other “basic” metabolic parameters, e.g., glucose and triglyceride concentrations. Our analysis showed that it was evaluated in 82% of the examples in Table 1. Interpretation of changes in total protein concentration is usually difficult. Increased levels of plasma proteins may be related to chronic inflammation. At the same time, decreased concentration may be, for example, a result of blood loss, e.g., internal bleeding due to organ damage or impaired plasma protein synthesis caused by hepatic dysfunction. Some data suggest that hypoproteinemia in fish after exposure to toxicants may be associated with changes in osmoregulation [7]. Velisek et al. [8] studied the influence of metribuzin on *Cyprinus carpio* and detected transudate in the body cavity of experimental individuals. In the opinion of these authors, the transudate in the body cavity resulted from the escape of proteins due to damage to renal tubular epithelial cells. This phenomenon led to marked hypoproteinemia in blood plasma.

Some researchers also analyzed the concentrations of albumin and globulin fractions. The results showed that no changes were most often observed (Table 1), and in the case of albumin, an increase or decrease occurred with approximately the same frequency (26% and 29%). In contrast, globulin more frequently decreased (Table 1). Plasma albumin is synthesized in the liver, and thus, the changes in its level are believed to reflect the organism’s hepatic health and nutritional status. Therefore, reduced albumin concentration may indicate hepatic dysfunction or/and malnutrition. Part of the globulin fraction consists of immune proteins produced by leukocytes; thus, globulin level is used as an indicator of immune status.

The concentration of triglycerides (Tgs) is a biochemical parameter strongly associated with diet, and thus, it is used to evaluate fish nutritional status [9,10]. It is also often measured in toxicological experiments. Tg level was measured in 52% of the cases in Table 1. An increase (35%) was noted almost six times more often than a decrease (6%). In most analyzed cases (59%), triglyceride concentration did not change significantly. An increase in Tg level is typical for some pesticides and antimicrobials, while a decrease was noted in the case of exposure to Al_2_O_3_ nanoparticles (Table 1). No information is available about interpreting changes in Tg concentration in fish blood. We can generally state that changes in Tg level result from lipid metabolism disorders related to hepatic dysfunction. Georgieva et al. [11] revealed that fatty degeneration in the liver of *Cyprinus carpio* resulted from exposure to various types of pesticides.

According to Zhu et al. [12], fish cholesterol metabolism is similar to that in mammals and other vertebrates: dietary cholesterol is absorbed by enterocytes, while endogenous cholesterol is synthesized in the liver. Cholesterol is bound to proteins and transported to peripheral tissues by blood. Total cholesterol is frequently evaluated in fish toxicological studies (68% of the cases listed in Table 1), and its changes are dependent on exposure strength, concentration, and duration, with no changes often observed at lower toxicant concentrations and short exposures. At higher exposure levels, total cholesterol concentrations usually increase. Blood cholesterol level is a biomarker of hepatic lipid metabolism.

According to Borchel et al. [13], when ATP levels decrease, creatine phosphate regenerates the ATP stores by transferring a high-energy phosphate to adenosine diphosphate (ADP). The products of this reversible reaction catalyzed by creatine kinases (CKs) are ATP and creatine. The results listed in Table 1 show no changes or increases in creatinine levels due to intoxication. These results show that creatinine concentration can be used as a fish intoxication biomarker. The physiological significance of this parameter in fish has not been finally established, but we can assume that blood creatinine concentration is an indicator of renal function.

Other biomarkers of nitrogen metabolism and excretory functions are ammonia, urea, and uric acid levels. Most teleost fishes are ammoniotelic, meaning ammonia is the main product of their amino acid catabolism [14]. Most ammonia is excreted from blood by diffusion through the gills directly to the water. Thus, the buildup of ammonia in the blood may indicate increased protein catabolism or impairment of gill excretory function. An increase in protein catabolism may be related to the activation of energetic metabolism caused by general stress or detoxification. However, most teleost species also produce various amounts of urea and uric acid that are excreted by the gills and kidneys. Some fish, such as marine elasmobranchs and very few teleosts, are obligatory or facultative ureotelics [15]. According to Wright and Land [16], urea production in teleosts may increase under alkaline or ammonia stress. The data in Table 1 show that these parameters were measured in some toxicological studies. In most cases, blood ammonia concentration in intoxicated fish showed no changes (57%), while the urea level was usually increased (63%). According to Napolitano et al. [17], urea production was a biomarker of nitrite stress in cichlids and zebrafish.

Few authors measured lactate and bilirubin concentrations as toxicity biomarkers in fish, and the results (Table 1) show that the concentrations of these compounds usually do not change (lactates) or increase (bilirubin). The lactate level increases due to glycolysis activation under hypoxic conditions [18] and is a physiological stress biomarker. According to Kalakonda et al. [19], bilirubin is a product of heme catabolism and results from erythrocyte hemolysis and hemoglobin, as well as from the turnover of other heme-containing proteins such as myoglobin, cytochromes, catalase, peroxidase, and tryptophan pyrrolase. Once bilirubin is released into the plasma, it is bound to albumin, which serves as its transporter, and taken up into the hepatocytes, where it is conjugated to glucuronic acid. Conjugation renders bilirubin water soluble and facilitates its renal and biliary excretion. Therefore, hyperbilirubinemia can be a biomarker of hematotoxicity and hemolytic action of chemicals, resulting in bilirubin overproduction or hepatotoxicity causing disturbances in bilirubin metabolism.

Toxicity-induced changes in the levels of most metabolic parameters in fish are often dependent on toxic agent concentration and exposure duration: no changes occur at the beginning of exposures and lower concentrations, and distinct alterations develop at higher exposure levels. Various parameters show different sensitivity to intoxication, and different directions of changes may occur. Among the parameters discussed, some are biomarkers of general physiological stress (glucose, lactates, and ammonia). In contrast, others indicate dysfunctions of particular organs, including the liver (total protein, albumins, triglycerides, cholesterol, and bilirubin) and kidney (creatinine), or suggest various metabolic disorders (nitrogenous metabolites).

**Table 1:** The effects of toxic agents on fish metabolic biomarkers.

GCS	Fish Species	Toxic Agent	Exp. dur. [d]	Concentration [mg/L]	No Change		Decrease	Increase	Reference
Em	*C. idella*	Selenium (as Na_2_SeO_4_)	10–30	0.798	–		TP, Glb	Glu, Alb, Tg, TC	Saha et al. [20]
				1.596	–		TP, Glb	Glu, Alb, Tg, TC	
Em	*C. punctata*	Selenium (as Na_2_SeO_4_)	10–30	1.466	Glu, Alb, TC		TP, Glb	Glu, Alb, Tg, TC	Saha et al. [20]
				2.932	–		TP, Glb	Glu, Alb, Tg, TC	
Em	*L. rohita*	Pb(NO_3_)_2_	4	6.80	Glu, Glb, Tg, TC, Lct		Alb	UAc, TB	Latif et al. [21]
Em	*O. mykiss*	Cadmium (as CdCl_2_ · H_2_O)	1–30	1 *	Glu, TP, TC		Tg, TC	Glu, TP	Heydarnejad et al. [22]
				3 *	Glu		Tg, TC	Glu, TP, Tg, TC	
Em	*O. niloticus*	Copper (as CuSO_4_ · 5H_2_O)	4–21	0.05	Glu, TP, TC		–	Glu	Firat et al. [2]
Em	*O. niloticus*	Lead [as Pb(NO_3_)_2_]	4–21	0.05	Glu, TP, TC		–	Glu	Firat et al. [2]
Em	*P. hypophthalmus*	Chromium (as K_2_Cr_2_O_7_)	4	10	Glu		–	–	Islam et al. [23]
				20	–		–	Glu	
				30	–		–	Glu	
				40	–		–	Glu	
F	*O. mykiss*	Propiconazole	7–30	0.2 *	Glu, TP, NH_3_		–	–	Li et al. [24]
				50 *	Glu, TP, NH_3_		–	Glu, NH_3_	
				500 *	Glu, TP, NH_3_		–	Glu, NH_3_	
H	*C. carpio*	Chwastox	1–10	1	Glu, TP, Tg, TC		–	–	Bojarski et al. [25]
				5	Glu, TP, Tg, TC		TP	–	
H	*C. carpio*	Glyphosate (as Roundup)	7	0.1	Glu		–	Tg, TC	Kondera et al. [26]
				0.5	Glu, Tg, TC		–	–	
				5.0	Glu, Tg, TC		–	–	
H	*C. carpio*	Roundup	1–10	1	Glu, TP, Tg, TC		–	TC	Bojarski et al. [27]
			1–3	5	Glu, TP, Tg, TC		–	Glu	
H	*C. gariepinus*	Oxyfluorfen	60	0.58	–		TP, Alb, Glb	Glu, Ur, Cr	Mansour et al. [28]
H	*L. rohita*	Glyphosate	4–12	0.5	Cr, Ur		–	–	Ghaffar et al. [29]
				0.6	Cr, Ur		–	–	
				0.7	Cr, Ur		–	Cr, Ur	
				0.8	–		–	Cr, Ur	
H	*O. niloticus*	Acetochlor	4	2.625	TP		–	Glu, Alb, Glb, TC, Cr, UAc	Fathy et al. [30]
H	*O. niloticus*	Bensulfuron methyl	4	2.50	Glu, TP, Glb, Cr		–	Alb, TC, UAc	Fathy et al. [30]
H	*O. niloticus*	Bentazon	4	36.00	Glu, Glb, Cr, UAc		TP	Alb, TC	Fathy et al. [30]
H	*O. niloticus*	Bispyribac sodium	4	0.800	Glu, TP, Cr, UAc		–	Alb, Glb, TC	Fathy et al. [30]
H	*O. niloticus*	Glyphosate (as Roundup)	14–28	0.6	–		TP, Alb, Glb	Glu, Cr, Ur	Abdelmagid et al. [31]
H	*O. niloticus*	Halosulfuron methyl	4	1.275	Glu, Glb, Cr, UAc		TP	Alb, TC	Fathy et al. [30]
H	*O. niloticus*	Propanil	14–56	0.22	Glu, TP, Tg, TC		–	TP	Yaji et al. [32]
				0.44	Glu, Tg, TC		TC	TP	
				0.87	Glu, Tg, TC		TC	Glu, TP	
H	*O. niloticus*	Quinclorac	4	11.250	Glu, TP, TC, Cr, UAc		–	Alb, Glb	Fathy et al. [30]
I	*C. carpio*	Chlorpyrifos	10–30	20 *	Glu, TP, Alb,		TP, Alb, Glb	Glu, Tg	Banaee et al. [3]
					Glb, Tg, TC				
				40 *	TP, Alb, Glb, TC		TP, Glb	Glu, Tg, TC	
I	*C. carpio*	Lindane	1	0.38	–		–	Glu, TP	Saravanan et al. [33]
			5–25	0.038	–		TP	Glu	
I	*C. carpio*	Profenofos	60	4.74 *	Alb		Glu, TP, Glb	Tg, TC, Cr, Ur	Abdel Rahman et al. [34]
I	*C. gariepinus*	Cypermethrin	4	0.025	TP, Tg		Glu, TC	–	Ojutiku et al. [35]
				0.050	Tg		Glu	TP, TC	
				0.075	Tg		Glu, TP	TC	
				0.100	Tg		Glu	TP, TC	
				0.125	–		Glu	TP, Tg, TC	
I	*C. gariepinus*	Imidacloprid (as Sunclopride 35% SC)	60	2.03 *	–		TP, Alb, Glb	Cr, Ur	Abdel Rahman et al. [36]
I	*C. idella*	Endosulfan (as Endosulfan 35 EC)	15–45	0.75 *	–		Glu, TP	TC	Bala [37]
				1 *	Glu		Glu, TP	TC	
I	*C. mrigala*	Diazinon (as Basudin 60 EC)	10–30	0.815	–		TP, Alb, Glb	Glu	Haider and Rauf [38]
				1.63	–		TP, Alb, Glb	Glu	
I	*C. punctata*	Malathion (commercial grade)	4–12	0.4	TC, Alb, Cr		Glu, Ur, UrN, TB	TP, Cr, Ur, UrN	Bharti and Rasool [39]
I	*L. rohita*	Pyriproxyfen	10–30	0.3	Glu, TP, Alb, Tg, TC, Cr, Ur	–		–	Naseem et al. [40]
	
				0.6	Glu, TP, Alb, Tg, TC, Cr, Ur	TP, Alb		Glu, Tg, TC, Cr, Ur	

				0.9	Glu, TP, Alb,	TP, Alb		Glu, Tg, TC, Cr, Ur	
					TC, Cr, Ur				
I	*L. rohita*	Thiamethoxam	3–5	0.5	Glu, TP, Glb, Tg, TC, Cr, Ur		–	–	Hussain et al. [41]
	
				1	Glu, TP, Glb, Tg, TC, Cr, Ur		–	–	
	
				1.5	Glu, TP, Glb, TC		TP, Glb	Glu, Tg, TC, Cr, Ur	
				2	–		TP, Glb	Glu, Tg, TC, Cr, Ur	
	
I	*O. mykiss*	Diazinon	60	0.287	Glu		–	–	Hajirezaee et al. [42]
I	*O. niloticus*	Cypermethrin	4–21	0.05 *	TP		–	Glu, TC	Firat et al. [2]
I	*R. quelen*	Fipronil	4	0.3	TP, Alb		–	–	Fredianelli et al. [43]
				0.4	TP, Alb		–	–	
N	*O. niloticus*	Al_2_O_3_-NPs	14	1	TC, Cr, UrN		Glu, Tg	–	Canli et al. [44]
				5	Tg, TC, Cr, UrN		Glu	–	
				25	TC		Glu, Tg	Cr, UrN	
N	*O. niloticus*	CuO-NPs	14	1	Tg, TC, Cr, UrN		Glu	–	Canli et al. [44]
				5	Tg, TC, Cr, UrN		Glu	–	
				25	Tg, TC, Cr, UrN		Glu	–	
N	*O. niloticus*	CuO-NPs	25	10	Ur		–	Cr	Abdel-Latif et al. [45]
				20	–		–	Ur, Cr	
				50	–		–	Ur, Cr	
O	*C. carpio*	Pyrene	35	10 *	TP, Alb		TC	Tg	Shirdel et al. [46]
				50 *	TP, Alb		TC	Tg	
				100 *	–		TP, TC, Alb	Tg	
O	*C. idella*	Ammonium acetate	n/a	9 **	Glu, TP, Alb, Glb, Tg, TC		–	–	Xing et al. [47]
O	*O. niloticus*	Methyl tert-butyl ether	27	2.5 ****	Alb, Tg		TP, Glb	Glu, TC, Cr	Banaee et al. [48]
				5 ****	Alb, Tg		TP, Glb	Glu, TC, Cr	
O	*P. fulvidraco*	Ammonium acetate	n/a	8 **	Alb, Tg, TC		Glu, TP, Glb	–	Zhang et al. [49]
Pd	*C. carpio*	Malachite green	7–14	0.2	Glu, TP, Tg, TC		–	–	Bojarski et al. [50]
Pd	*C. carpio*	Methylene blue	7–14	2	Glu, TP, Tg, TC		–	TP	Bojarski et al. [50]
Pd	*C. gariepinus*	Clotrimazole (commercial formulation)	1–21	1.94	Glu, TP		TP	Glu	Melefa et al. [51]
				3.89	–		Glu, TP	Glu	
				7.76	Glu		Glu, TP	Glu	
Pd	*H. nobilis*	Triclosan	5–15	1	TP, Tg, TC, Cr, Ur		–	–	Akram et al. [52]
				1.5	TP, Tg, TC, Cr, Ur		TP	Tg, TC, Cr, Ur	
				2.5	TP, Tg, TC, Cr, Ur		TP	Tg, TC, Cr, Ur	
Pd	*L. rohita*	CuSO_4_ · 5H_2_O	4	3.15	Glu, Glb, Lct		Alb	Tg, TC, UAc, TB	Latif et al. [21]
Pd	*P. fluviatilis*	Propiscin (etomidate)	3 min	1 ***	TP, Alb, Glb, Cr, NH_3_, TB		–	Glu, Lct	Rożyński et al. [53]
		
				2 ***	TP, Alb, Glb, Cr, TB		–	Glu, NH_3_,	
								Lct	
			10 min	1 ***	Glu, TP, Alb,		–	Lct	
					Glb, Cr, NH_3_, TB				
				2 ***	Glu, TP, Alb,		–	–	
					Glb, Cr, NH_3_,				
					TB, Lct				
Pd	*V. vimba*	2-phenoxy- ethanol	10 min	0.4 ***	TP, Alb, Glb, Tg, Lct		–	Glu, NH_3_	Lepic et al. [54]
Pd	*V. vimba*	MS 222	10 min	100	Glu, TP, Alb, Glb, Tg, NH_3_, Lct		–	–	Lepic et al. [54]
Pd	*V. vimba*	Propiscin (etomidate)	10 min	1 ***	TP, Alb, Glb, Tg, NH_3_, Lct		–	Glu	Lepic et al. [54]

* µg/L; ** µmol/g fish (injection); *** mL/L; **** µL/L. GCS—group of chemical substances; Em—metals and other elements; F—fungicides; H—herbicides; I—insecticides; N—nanoparticles; O—other substances; Pd—pharmaceuticals and disinfectants. Exp. dur.—exposure duration; d—days; n/a—not applicable; NPs—nanoparticles. *C. carpio*—*Cyprinus carpio*, *C. gariepinus*—*Clarias gariepinus*, *C. idella*—*Ctenopharyngodon idella*, *C. mrigala*—*Cirrhinus mrigala*, *C. punctata—Channa punctata*, *H. nobilis*—*Hypophthalmichthys nobilis*, *L. rohita*—*Labeo rohita*, *O. mykiss—Oncorhynchus mykiss*, *O. niloticus*—*Oreochromis niloticus*, *P. fluviatilis*—*Perca fluviatilis*, *P. fulvidraco*—*Pelteobagrus fulvidraco*, *P. hypophthalmus*—*Pangasianodon hypophthalmus*, *R. quelen—Rhamdia quelen*, *V. vimba*—*Vimba.* Glu—glucose; TP—total protein; Alb—albumins; Glb—globulins; Tg—triglycerides; TC—total cholesterol; Cr—creatinine; Ur—urea; UrN—urea nitrogen; UAc—uric acid; NH_3_—ammonia; TB—total bilirubin; Lct—lactate.

## 2. The Effects of Toxic Agents on Fish Tissue Damage Biomarkers

The parameters used to assess the impact of toxic substances on fish also include enzymatic biomarkers [55]. Some metabolic enzymes, such as aminotransferases, are considered to be sensitive indicators of hepatotoxicity since the fish liver is the main depot for bioaccumulation of many types of xenobiotics and also the main detoxification organ [56]. Alanine aminotransferase (also called alanine transaminase; ALT) is an enzyme that catalyzes the transfer of an amino group from alanine to α-ketoglutarate (2-oxoglutarate), forming pyruvate and glutamate; the reaction is reversible. Aspartate aminotransferase (also called aspartate transaminase; AST) catalyzes the reversible reaction of transferring the amino group from aspartate to α-ketoglutarate with the formation of oxaloacetate and glutamate. ALT and AST are localized mainly in the liver, heart, gills, kidneys, and muscles [57]. An increase in plasma ALT or AST activity is caused by the leakage of these enzymes from the cells into the interstitial fluid and the bloodstream due to cell membrane disruption. Therefore, they are called “leakage enzymes” and are used as tissue (mainly hepatic) damage biomarkers. An increase in plasma or serum ALT activity appears to reflect liver disease and is more specific for liver dysfunctions than elevated AST activity [57]. Thus, the measurement of ALT activity is the gold standard for the detection of liver injury in clinical chemistry [58]. According to Savari et al. [59], in the absence of severe muscle necrosis, a significant increase in serum ALT level results only from hepatocyte damage; these authors stated that the magnitude of elevation is proportional to the number of affected hepatocytes. Increased plasma AST activity was noted in Atlantic salmon (*Salmo salar*) with nephrocalcinosis, indicating that this enzyme is not highly specific for liver disorders [60]. Matsche and Gibbons [61] listed that elevated blood AST activity may indicate stress, infection, liver or kidney dysfunction, and increased metabolism of amino acids. Alanine and aspartate are major glucogenic precursors and important energy substrates in fish [62], and therefore, increased AST activity noted in females of some fish species may reflect an increased energy demand related to egg production [61]. On the other hand, variations in the activities of enzymes measured in blood, such as ALT and AST, may be associated with fish age, habitats, and diets [61]. Moreover, they can result from different sampling techniques and blood analysis methods [63]. Intra- and interspecific differences in ALT and AST activity appear significant. Tripathi et al. [64] demonstrated that serum ALT activity measured in *Cyprinus carpio* was 28–35 U/L while AST activity was 71–98 U/L. However, according to Kondera et al. [65] the activity of aminotransferases in plasma obtained from *Cyprinus carpio* blood was 74.0 ± 13.8 U/L (ALT) and 67.0 ± 8.9 U/L (AST). ALT activity measured in the blood plasma of *Rhamdia quelen* was 1.87 ± 0.32 and 5.73 ± 1.51 U/L, while the AST level was 11.98 ± 1.68 and 21.31 ± 3.07 U/L [66]. Mahamood et al. [67] observed that in *Labeo rohita*, serum ALT activity was 8.31 ± 0.1 while serum AST activity was 4.87 ± 0.01 U/L. Wnęk-Auguścik et al. [6] analyzed blood sampled from *Acipenser baerii* and revealed that ALT and AST determined in plasma were, respectively, 50.2 ± 16.3 and 321.0 ± 131.6 U/L. In the case of humans, the magnitude of aminotransferase alteration is classified as “mild” (<5 times the upper reference limit), “moderate” (5–10 times the upper reference limit), or “marked” (>10 times) [68]. In fish toxicology, ALT and AST activity are commonly measured (in plasma or serum of fish blood), and such an approach could also be used, even despite a lack of commonly accepted reference values, using the values obtained for a control or reference group of fish instead. Our analysis showed that no change (52%) and an increase (42%) were often noted in the case of ALT. At the same time, a decrease was a rare phenomenon—it occurred in 6% of the examples listed in Table 2 (decreased activity was observed in the case of some herbicides and the insecticide malathion). An increase in plasma or serum ALT activity is usually interpreted as evidence of the hepatotoxic effect of chemicals, while a decrease is difficult to explain. Increased ALT activity was noted in fish exposed to heavy metals, pesticides (especially insecticides), nanoparticles, and pharmaceuticals (Table 2). To the best of our knowledge, in experimental toxicology, AST activity measured in fish blood is determined for a similar purpose as in the case of ALT, i.e., mainly to detect liver dysfunction. Our analysis showed that an increase was the most often noted result (57%), while a decrease was rare (9% of the examples in Table 2). In fish toxicology research, the amount of blood available for analysis may be a limitation. Therefore, when it is impossible to determine the activity of both ALT and AST, we recommend testing ALT activity due to the higher specificity of this indicator for liver dysfunction compared to AST.

Many toxicological studies are performed using embryos and larvae (usually *Danio rerio*), and ALT and AST activities are measured in the whole-body homogenate. For example, Zhang et al. [69] observed dose-dependent increases in these parameters in *Danio rerio* larvae exposed to isopsoralen (an anti-inflammatory agent). Martins et al. [70] treated *Danio rerio* larvae with 2,4-D herbicide and measured enzyme activities in the obtained whole-body supernatant. ALT, AST, and ALP activity markedly increased at all concentrations, as did LDH at most herbicide concentrations.

Alkaline phosphatase (ALP) was identified as one of the first clinically relevant enzymes [57]. It plays an important role in phosphate hydrolysis and membrane transfer and is involved in the transphosphorylation of various biomolecules in alkaline pH. The activity of ALP is essential for cell growth, migration, and apoptosis [71]. According to Kulkarni [72], the exact metabolic function of ALP in fish is not fully clarified, but it appears that this enzyme is associated with calcification of bones and is probably involved in lipid transport in the intestine. The fish liver synthesizes more ALP than other organs [57], but high levels of this enzyme were also found in kidney tubules, intestinal epithelium, and osteoblasts [72]. ALP is a non-specific marker used to diagnose liver and bone damage. Moreover, since this enzyme may play an essential role in providing energy to stressed cells, its activity can be used to assess cellular stress [71]. According to Shahsavani et al. [57], a highly increased amount of ALP in the blood may result from liver or skeletal disorders. Interestingly, some data indicate that plasma ALP has potential as a predictive diagnostic tool in evaluating the nutritional status of European seabass (*Dicentrarchus labrax*) [73]. Our analysis of the results of measurements of blood ALP activity conducted in various toxicological experiments (Table 2) showed that no change (47%) and an increase (45%) were often noted; a decrease in ALP activity occurred rarely. Increased ALP levels were typical for exposures to various heavy metals and pesticides (Table 2). As ALP is not liver-specific, we believe the measurement of its plasma or serum activity should not be used as the sole marker of hepatotoxicity and should only have auxiliary significance in fish toxicology.

Lactate dehydrogenase (LDH) is an essential enzyme of the anaerobic metabolic pathway; it is responsible for reversible conversion of pyruvate to lactate. Under stress conditions, the anaerobic pathway is activated, high lactate levels are produced, and high LDH activities are observed [74]. Gupta [75] demonstrated that LDH activity in *Channa punctatus* in the tested organs differed; it was the highest in the gills, brain, and muscles, visibly lower in the liver and heart, and the lowest in the kidney. Measurements of LDH activity are used to detect tissue and organ damage in fish and some aquatic invertebrates [76,77]. For example, exposure of *Channa punctatus* to tannery wastewater increased serum LDH activity [78]. Moreover, *Salmo trutta* and *Oncorhynchus mykiss* exposed to microcystin-LR exhibited elevated plasma LDH activity [79]. It was also demonstrated that treatment with nitrites caused an increase in serum LDH activity in fingerlings of *Catla catla, Labeo rohita*, and *Cirrhinus mrigala* [80]. Our analysis showed that blood LDH activity is much less often determined in fish toxicology than ALT, AST, and ALP levels (Table 2). An increase (52%) was the most often noted result, while a decrease was rarely observed (8%). LDH activity may be useful as an additional general stress biomarker.

**Table 2:** The effects of toxic agents on fish tissue damage biomarkers.

GCS	Fish Species	Toxic Agent	Exp. dur. [d]	Concentration [mg/L]	No Change	Decrease	Increase	Reference
Em	*L. rohita*	Pb(NO_3_)_2_	4	6.80	ALT, AST, LDH	–	ALP	Latif et al. [21]
Em	*M. cephalus*	CuO	21	0.79 1.57	– –	ALP ALP	ALT, AST, LDH ALT, AST, LDH	Akbary et al. [81]
Em	*O. mykiss*	Cadmium (as CdCl_2_·H_2_O)	1–30	1 * 3 *	ALT, ALP ALT, AST, ALP	AST –	ALT, AST, ALP ALT, AST, ALP	Heydarnejad et al. [22]
Em	*O. niloticus*	Copper (as CuSO_4_·5H_2_O)	4–21	0.05	ALP, LDH	–	ALT, AST, ALP, LDH	Firat et al. [2]
Em	*O. niloticus*	Lead [as Pb(NO_3_)_2_]	4–21	0.05	ALP, LDH	–	ALT, AST, ALP, LDH	Firat et al. [2]
F	*O. mykiss*	Propiconazole	7–30	0.2 * 50 * 500 *	ALT, AST, LDH ALT, AST, LDH ALT, AST, LDH	– – –	– LDH ALT, AST, LDH	Li et al. [24]
H	*C. carpio*	Chwastox	1–10	1 5	ALT ALT	– ALT	– –	Bojarski et al. [25]
H	*C. carpio*	Roundup	1–10 1–3	1 5	ALT ALT	– –	– –	Bojarski et al. [27]
H	*C. carpio*	Paraquat (as Gramoxone)	21	0.2 0.4	AST –	– –	ALT, ALP, LDH ALT, AST, ALP, LDH	Nematdoost Haghi and Banaee [82]
H	*C. gariepinus*	Oxyfluorfen	60	0.58	–	–	ALT, AST, ALP	Mansour et al. [28]
H	*H. bidorsalis*	Glyphosate	30	0.16 0.32 0.48	ALP ALP –	ALT, AST ALT, AST ALT, AST, ALP	– – –	Inyang et al. [83]
H	*L. rohita*	Glyphosate	4–12	0.5 0.6 0.7 0.8	ALT, AST, ALP ALT, AST, ALP – –	– – – –	– – ALT, AST, ALP ALT, AST, ALP	Ghaffar et al. [29]
H	*O. niloticus*	Acetochlor	4	2.625	ALT	–	AST, ALP	Fathy et al. [30]
H	*O. niloticus*	Bensulfuron methyl	4	2.50	ALT	–	AST, ALP	Fathy et al. [30]
H	*O. niloticus*	Bentazon	4	36.00	ALT, ALP	–	AST	Fathy et al. [30]
H	*O. niloticus*	Bispyribac sodium	4	0.800	ALT	–	AST, ALP	Fathy et al. [30]
H	*O. niloticus*	Glyphosate (as Roundup)	14–28	0.6	–	–	ALT, AST	Abdelmagid et al. [31]
H	*O. niloticus*	Halosulfuron methyl	4	1.275	ALT, ALP	–	AST	Fathy et al. [30]
H	*O. niloticus*	Propanil	14–56	0.22 0.44 0.87	ALT, AST ALT ALT	– – –	AST ALT, AST ALT, AST	Yaji et al. [32]
H	*O. niloticus*	Quinclorac	4	11.250	ALT, AST	–	ALP	Fathy et al. [30]
I	*C. carpio*	Chlorpyrifos	10–30	20 * 40 *	ALT, AST, ALP, LDH ALT, ALP, LDH	– –	AST, LDH ALT, AST, LDH	Banaee et al. [3]
I	*C. carpio*	Profenofos	60	4.74 *	–	–	ALT, AST, ALP, LDH	Abdel Rahman et al. [34]
I	*C. gariepinus*	Cypermethrin	4	0.025 0.050 0.075 0.100 0.125	ALT, ALP ALP ALT, ALP ALP ALP	AST AST AST AST –	– ALT – ALT ALT, AST	Ojutiku et al. [35]
I	*C. gariepinus*	Deltamethrin (as Butox)	2	0.75 *	–	–	ALT, AST	Amin and Hashem [84]
I	*C. gariepinus*	Imidacloprid (as Sunclopride 35% SC)	60	2.03 *	–	–	ALT, AST	Abdel Rahman et al. [36]
I	*C. mrigala*	Diazinon (as Basudin 60 EC)	10–30	0.815 1.63	ALT, AST, LDH –	LDH LDH	ALT, AST ALT, AST	Haider and Rauf [38]
I	*C. punctata*	Malathion (commercial grade)	4–12	0.4	–	ALT, AST, ALP	–	Bharti and Rasool [39]
I	*L. rohita*	Pyriproxyfen	10–30	0.3 0.6 0.9	ALT, AST, ALP, LDH ALT, AST, ALP, LDH –	– – –	– ALT, AST, ALP, LDH ALT, AST, ALP, LDH	Naseem et al. [40]
I	*L. rohita*	Thiamethoxam	3–5	0.5 1 1.5 2	ALT, AST, ALP, LDH ALT, AST, ALP, LDH – –	– – – –	– – ALT, AST, ALP, LDH ALT, AST, ALP, LDH	Hussain et al. [41]
I	*O. niloticus*	Cypermethrin	4–21	0.05 *	–	–	ALT, AST, ALP, LDH	Firat et al. [2]
I	*R. quelen*	Fipronil	4	0.3 0.4	ALP –	– –	ALT, AST ALT, AST, ALP	Fredianelli et al. [43]
N	*O. niloticus*	Ag-NPs	60	1.98	–	–	ALT, AST	Farag et al. [85]
N	*O. niloticus*	Al_2_O_3_-NPs	14	1 5 25	ALT, AST, ALP ALT, AST, ALP ALT, AST, ALP	– – –	– – –	Canli et al. [44]
N	*O. niloticus*	CuO-NPs	14	1 5 25	ALT, AST, ALP ALT, AST, ALP ALT, AST, ALP	– – –	– – –	Canli et al. [44]
N	*O. niloticus*	CuO-NPs	25	10 20 50	– – –	– – –	ALT, AST, ALP ALT, AST, ALP ALT, AST, ALP	Abdel-Latif et al. [45]
N	*O. niloticus*	TiO_2_-NPs	14	1 5 25	ALT, AST, ALP ALT, AST, ALP ALT, AST, ALP	– – –	– – –	Canli et al. [44]
O	*C. carpio*	Pyrene	35	10 * 50 * 100 *	ALT, AST, ALP ALT, AST –	– – ALT	– ALP AST, ALP	Shirdel et al. [46]
O	*C. idella*	Ammonium acetate	n/a	9 **	–	–	ALP	Xing et al. [47]
O	*O. niloticus*	Methyl tert-butyl ether	27	2.5 **** 5 ****	ALT –	– –	AST, ALP, LDH ALT, AST, ALP, LDH	Banaee et al. [48]
O	*P. fulvidraco*	Ammonium acetate	n/a	8 **	–	ALP	–	Zhang et al. [49]
Pd	*C. carpio*	Malachite green	7–14	0.2	ALT	–	–	Bojarski et al. [50]
Pd	*C. carpio*	Methylene blue	7–14	2	ALT	–	–	Bojarski et al. [50]
Pd	*C. gariepinus*	Clotrimazole (commercial formulation)	1–21	1.94 3.89 7.76	ALT, AST, ALP ALT, AST –	– – –	ALT, AST, ALP ALT, AST, ALP ALT, AST, ALP	Melefa et al. [51]
Pd	*C. gariepinus*	Diclofenac	42	1.57 3.14 6.28	– – –	– – –	ALT, AST, LDH ALT, AST, LDH ALT, AST, LDH	Ajima et al. [86]
Pd	*H. nobilis*	Triclosan	5–15	1 1.5 2.5	ALT, AST ALT, AST ALT, AST	– – –	– ALT, AST ALT, AST	Akram et al. [52]
Pd	*L. rohita*	CuSO_4_·5H_2_O	4	3.15	ALT, AST, ALP	–	LDH	Latif et al. [21]
Pd	*P. fluviatilis*	Propiscin (etomidate)	3 min 10 min	1 *** 2 *** 1 *** 2 ***	ALT, AST, ALP ALT, AST, ALP ALT, AST, ALP ALT, AST, ALP	– – – –	– – – –	Rożyński et al. [53]
Pd	*V. vimba*	2-phenoxy- ethanol	10 min	0.4 ***	ALT, ALP, LDH	–	AST	Lepic et al. [54]
Pd	*V. vimba*	MS 222	10 min	100	ALT, AST, ALP	LDH	–	Lepic et al. [54]
Pd	*V. vimba*	Propiscin (etomidate)	10 min	1 ***	ALT, ALP, LDH	AST	–	Lepic et al. [54]

* µg/L; ** µmol/g fish (injection); *** mL/L; **** µL/L. GCS—group of chemical substances; Em—metals and other elements; F—fungicides; H—herbicides; I—insecticides; N—nanoparticles; O—other substances; Pd—pharmaceuticals and disinfectants. Exp. dur.—exposure duration; d—days; n/a—not applicable; NPs—nanoparticles. *C. carpio*—*Cyprinus carpio*, *C. gariepinus*—*Clarias gariepinus*, *C. idella*—*Ctenopharyngodon idella*, *C. mrigala*—*Cirrhinus mrigala*, *C. punctata—Channa punctata*, *H. bidorsalis—Heterobranchus bidorsalis*, *H. nobilis*—*Hypophthalmichthys nobilis*, *L. rohita*—*Labeo rohita*, *M. cephalus—Mugil cephalus*, *O. mykiss—Oncorhynchus mykiss*, *O. niloticus*—*Oreochromis niloticus*, *P. fluviatilis*—*Perca fluviatilis*, *P. fulvidraco*—*Pelteobagrus fulvidraco*, *R. quelen—Rhamdia quelen*, *V. vimba*—*Vimba vimba.* ALT—alanine aminotransferase; AST—aspartate aminotransferase; ALP—alkaline phosphatase; LDH—lactate dehydrogenase.

## 3. The Effects of Toxic Agents on Hormone Levels in Fish

Many aquatic pollutants may alter the functions of the vertebrate endocrine system. According to the definition proposed by the World Health Organization (WHO) and International Programme on Chemical Safety (IPCS) [87] and widely accepted scientifically, endocrine disrupting chemicals (EDCs) are exogenous substances or mixtures that alter function(s) of the endocrine system and consequently cause adverse health effects in an intact organism, or its progeny, or (sub)populations. Many endocrine-disrupting chemicals may cause transgenerational effects due to the interference with animal reproductive function and hormone signaling [88].

Methods of evaluation of endocrine disruption in fish are diverse, including reproduction, growth, and development parameters, molecular analyses of expression of genes encoding hormones or their receptors, or direct measurements of hormone levels in blood plasma or serum. Our analysis of the results (Table 3) focuses on the latter as a strictly biochemical method. However, studies of endocrine toxicity are often performed on early developmental stages of fish (embryos and larvae) that are very sensitive to environmental impacts. In such cases, the whole organism is used for biochemical analysis. Such studies are relatively common, so we added these types of experiments to Table 3. Embryonic and larval studies mainly evaluate toxic agents on thyroid hormone (T3 and T4) levels, as these hormones play a key role in fish early development and metamorphosis [89]. Measuring embryonic and larval hormone levels is also essential in evaluating the transgenerational endocrine effects of parental exposures. According to Robaire et al. [90], teleost fishes are commonly used as models for the multi- and transgenerational effects of EDCs.

Table 3 includes 50 examples of various chemicals’ effects on fish hormone levels. The thyroid hormones T3 (triiodothyronine) and T4 (thyroxine) were studied the most frequently (58% each), followed by cortisol (30%), 17β-estradiol or estrone (28%), testosterone or 11-ketotestosterone (22%), and growth hormone (20%). Some authors measured the levels of insulin-like growth factor (IGF) (12%), the gonadotropins FSH and LH (12% each), corticotropin-releasing hormone (CRH), and thyrotropin (TSH) (4% each). The glucagon level and adrenocorticotropic hormone (ACTH) concentration were analyzed least often (2% each).

The results showed that the studied compounds and elements disrupted thyroid functions, usually inhibiting T3 and T4 secretion; however, sometimes different effects on the levels of each hormone occurred, while some toxic agents caused hyperthyroidism. In some cases, a decrease in T3 was accompanied by an increase in T4, which indicates the adverse effects of chemicals on deiodinases. According to Deal and Volkoff [91], in all vertebrates, including fish, T3 and T4 are key hormones controlling numerous physiological processes, including growth, development/morphogenesis, and metabolism. Secretion of vertebrate T3 and T4 is regulated by the hypothalamus–pituitary–thyroid (HPT) axis. Thyrotropin-releasing hormone (TRH) secreted by hypothalamic neurosecretory cells stimulates the anterior pituitary to produce thyrotropin (TSH) that acts on the thyroid, enhancing the production of T3 and T4 that reciprocally inhibit TSH and TRH release. However, it is supposed that in some fish species, TSH may be more stimulated by corticotropin-releasing hormone (CRH) than TRH. T3 and T4, secreted from thyroid follicles, undergo peripheral regulation to exert their effects. Iodothyronine deiodinases are peripheral tissue enzymes that convert T4 (prohormone) to T3 (active hormone) and regulate their availability and disposal. The results shown in Table 3 indicate that the levels of thyroid hormones are sensitive biomarkers of intoxication, and additional measurements of TRH and TSH may help to explain the mechanisms of thyrotoxicity.

Various compounds showed different effects on the levels of sex steroids in fish; in most cases, a suppression was observed. However, some chemicals showed estrogenic effects, such as increasing estrogen levels and decreasing testosterone concentrations. In most cases, the hormonal reaction of females and males to intoxication was different. In vertebrates, the hypothalamus–pituitary–gonadal (HPG) axis regulates maturation and reproduction: the hypothalamus secretes the gonadotropin-releasing hormone (GnRH) that stimulates the pituitary to release gonadotropins, luteinizing hormone (LH) and follicle-stimulating hormone (FSH), which act on gonads and control oogenesis, spermatogenesis, and synthesis of sex steroid hormones [91]. Toxicity-induced reduction of sex steroid levels is often mediated by oxidative stress, and accompanied by inflammation and apoptosis in the gonads that lose their physiological functions [92]. Oxidative stress was proved to disrupt sex steroid hormone synthesis by dysregulating gene expression that caused reproductive toxicity in both male and female zebrafish exposed to perfluorobutane sulfonate [93]. Evidence of the effects of toxic agents on sex steroid levels and HPG regulation makes these hormones valuable biomarkers of possible reproductive toxicity.

Cortisol is a commonly used primary stress reaction biomarker, and many toxic compounds induce nonspecific physiological stress in fish; thus, the use of this biomarker in toxicology is well justified. The data shown in Table 3 indicate that cortisol level changes were related to the concentrations of toxic agents and duration of exposures, and in 100% of cases when a cortisol reaction occurred, it was an increase. Thus, cortisol measurement may be considered a reliable and sensitive (but nonspecific) biomarker of toxicant exposure. According to Lemos et al. [94], fish are more sensitive to stressors than many other vertebrates, making them ideal candidates for stress response assessments in different fields, e.g., ecotoxicology. The stress reaction includes activation of the HPA axis (hypothalamic–pituitary–adrenal), the action of which starts with the release of the hypothalamic polypeptide corticotropin-releasing hormone (CRH). CRH stimulates the secretion of the adrenocorticotrophic hormone (ACTH) from the anterior pituitary gland. ACTH activates inter-renal tissue in fish to synthesize and release cortisol [94]. CRH and ACTH levels are very rarely measured in fish, and it is difficult to conclude if their measurements add more information about the stress reaction than cortisol itself.

Growth hormone (GH) and insulin-like growth factor (IGF) are the primary regulators of somatic growth in vertebrates [95]. According to Reinecke et al. [96], growth hormone (GH) produced by the pituitary gland in teleosts acts directly by binding to a transmembrane receptor in target tissues and participates in many physiological processes including osmoregulation, nutrient metabolism, tissue growth, reproduction, immune response, and behavior. Insulin-like growth factors, IGFs, are hepatic peptides structurally related to insulin. IGFs are mainly involved in the development and growth of many organs as mediators of GH. In fish, transcription of the IGF-1 gene in the liver and other tissues is controlled by the action of GH, while the expression of IGF-2 depending on GH is less evidenced [97]. The IGF-1 levels in fish are positively related to the growth rate and growth-promoting environmental conditions, including temperature, food abundance, and quality, while less is known about IGF-2 and the recently discovered gonad-specific IGF-3. These relationships make IGF-1 a promising biomarker of environmental impacts on fish, e.g., growth impairment due to intoxication.

Some endocrine disruptors (EDs) may exert intergenerational effects in the offspring of exposed individuals. The intergenerational effects of EDs are not limited to epigenetic changes such as DNA methylation, histone modification, or expression of non-coding RNA. They also involve a direct transfer of compounds from females to their eggs [98]. Xu et al. [99] evaluated the maternal transfer risk of uranium in zebrafish. Exposure of mature female zebrafish to 2 and 20 ng/g of uranium-spiked food for 28 days resulted in uranium bioconcentration in females and their embryos, accompanied by a significant decrease in the levels of triiodothyronine (T3) in both the adults and the embryos. Teng et al. [100] evaluated the development of offspring after propiconazole exposure of zebrafish parents and reported an increase in T3 levels in the hatched larvae. According to Wang et al. [101], parental exposure of zebrafish to tris(2-chloroethyl) phosphate (TCEP) resulted in developmental toxicity, including declined thyroxin (T4) levels, indicating disruption of the maternal transfer of thyroid hormones to the offspring.

Additionally, the expression of several genes related to the hypothalamic–pituitary–thyroid axis was significantly modified in the larvae, indicating intergenerational developmental toxicity and thyroid disruption. Li et al. [102] subjected parental zebrafish to tris (1,3-dichloro-2-propyl) phosphate during early life. The results showed thyroid disruption: an increase in T3 and a decrease in T4 in the adult females after exposure. Neurodevelopmental disturbances followed similar alterations in the spawned eggs but not larvae in offspring. The authors stress two possible mechanisms of these disturbances: altered T3 and T4 levels during embryonic development, inherited from maternal zebrafish, and epigenetic modifications in larvae. The thyroid hormone levels in parental fish and their offspring are a reliable biomarker of exposure and the action of EDs. They provide essential and valuable information, since maternal thyroid hormone transfer plays a key role in the early development of the central nervous system. Thyroid hormones also affect the reproductive system, including gamete development, sex steroid synthesis, and sexual behavior, and there is evidence of a crosstalk between the HPT and HPG axes in fish [91]. According to Luo et al. [103], the combined exposure of zebrafish to microplastics and arsenic resulted not only in reproductive toxicity in exposed individuals (reduced gonadal indices, disturbed oocyte development, and disrupted sex hormone levels) due to oxidative stress but also increased embryo mortality rate, reduced hatching rate, and reduced larval heart rate. These findings provide evidence that co-exposure to MPs and arsenic damages the reproductive system and adversely affects offspring growth and development.

The data from various studies concerning toxic agents’ effects on fish hormone levels showed that many xenobiotics induce hormonal imbalances that may result in considerable developmental, behavioral, metabolic, and reproductive disturbances and intergenerational adverse effects. It seems that hormonal endpoints such as measurements of thyroid hormones, sex steroids, cortisol, and insulin-like growth factors are sensitive but nonspecific biomarkers of intoxication in fish. The information on endocrine toxicity can be broadened by including measurements of appropriate releasing and tropic hormones to evaluate toxic effects on hormone secretion regulatory axes.

**Table 3:** The effects of toxic agents on hormone levels in fish (↑ increase, ↓ decrease, – no change).

GCS	Fish Species	Toxic Agent	Concentration [mg/L]	Exp. dur. [d]	Hormone Activity	Reference
Em	*D. rerio* embryos/larvae	Pb(CH_3_CO_2_)_2_·3H_2_O	5–10 20	5	GH– IGF↓ GH↑ IGF↓	Yan et al. [104]
Em	*D. rerio* embryos/larvae	UO_2_^2+^ (as UO_2_(NO_3_)_2_·6H_2_O	2 * 20 * 100 *	5	T3– T4– T3↓ T4↑ T3↓ T4–	Xu et al. [105]
Em	*D. rerio* embryos/larvae	Cadmium (as CdCl_2_·2.5H_2_O)	10 * 100 * 1000 *	5	T3– T4– T3↓ T4↑ T3↓ T4↑	Zhong et al. [106]
Em	*D. rerio* embryos/larvae	Mercury (as HgCl_2_)	0.1 * 1 * 10 *	5	T3– T4↑ T3– T4↑ T3↑ T4↑	Zhong et al. [106]
Em	*D. rerio* embryos/larvae	Copper (as CuSO_4_·5H_2_O)	1.5 * 15–150 *	5	T3– T4– T3↓ T4↑	Zhong et al. [107]
Em	*D. rerio* embryos/larvae	Zinc (as ZnSO_4_·7H_2_O)	20 * 200 * 2000 *	5	T3– T4– T3– T4↑ T3↓ T4↑	Zhong et al. [107]
Em	*D. rerio* embryos/larvae	Cadmium (as CdCl_2_)	0.05–0.5 *** 1 ***	4	T3– T4– T3↑ T4↓	Di Paola et al. [108]
Em	*D. rerio* embryos/larvae	ZnCl_2_	0.1	5	GH– FSH– LH↓ E–	Liu et al. [109]
Em	*H. molitrix*	Mercury (as HgCl_2_)	0.05–0.5 * 5 * 50 *	7–28 7 14 28 7 14 28	GH– IGF– T3– T4– GH– IGF– T3– T4↓ GH– IGF– T3↓ T4↓ GH– IGF– T3↓ T4– GH↓ IGF↓ T3– T4↓ GH↓ IGF– T3↓ T4↓ GH↓ IGF– T3↓ T4–	Pu et al. [110]
Em	*M. cephalus*	NiCl_2_	5 * 10 * 15 *	4	C↑ T3↓ T4↑ C↑ T3↓ T4↓ C↑ T3↓ T4↓	Jasim et al. [111]
Em	*P. hypophthalmus*	Manganese (as Mn(CH_3_CO_2_)_2_	110–114	4	C↑	Kumar et al. [112]
F	*D. rerio*	Difenoconazole	5–500 *	7	♀ GH↑ IGF↑ ♂ GH↑ IGF↑	Teng et al. [113]
F	*D. rerio*	Tebuconazole	0.4 0.8 1.6	21	♀♂ E– T– ♀ E– T– ♂ E– T↓ ♀ E↓ T↓ ♂ E– T↓	Yan et al. [114]
H	*C. gariepinus*	Oxyfluorfen	0.58	60	♂ E↑ T↓ FSH↑ LH↓	Mansour et al. [28]
H	*D. rerio* embryos/larvae	Glyphosate	0.7–7 35	5	GH– IGF– GH↓ IGF↓	Liu et al. [115]
H	*L. rohita*	Butachlor (as Shaktiman^®^)	12.42–62.1 *	1 2–3	C– C↑	Kumar et al. [116]
H	*M. salmoides*	Glyphosate	0.5–10	21	C– T↓ E↓	De Maria et al. [117]
H	*M. salmoides*	Glyphosate (as Rodeo^®^)	0.5–10	21	C– T↓ E↓	De Maria et al. [117]
H	*P. major*	Diuron	0.1 *	30–60	C– E– T–	Nam et al. [118]
			1 *	30	C↑ E– T–	
				60	C– E– T–	
			10 *	30	C– E– T–	
				60	C↑ E↓ T↓	
H	*S. schlegelii*	Diuron	0.1 *	30–60	C– E– T–	Nam et al. [118]
			1 *	30	C– E– T–	
				60	C↑ E↓ T–	
			10 *	30	C↑ E↓ T↓	
				60	C↑ E– T↓	
I	*L. catla*	Cypermethrin	0.14–0.7 *	30	E↓ T↓	Ganguly et al. [119]
I	*O. mykiss*	Diazinon	0.287	60	T3– T4↓ C↑	Hajirezaee et al. [42]
N	*O. niloticus*	ZnO-NPs	1.14	28	T3↓ T4↓	Abou-Zeid et al. [120]
N	*O. niloticus*	Ag-NPs	1.98	60	GH↓ T3– T4– G–	Farag et al. [85]
N	*O. niloticus*	Al_2_O_3_-NPs	1 5 25	14	C– T3– T4– C– T3– T4↓ C↑ T3– T4–	Canli et al. [44]
N	*O. niloticus*	CuO-NPs	1 5 25	14	C– T3– T4– C– T3– T4– C– T3– T4–	Canli et al. [44]
N	*O. niloticus*	TiO_2_-NPs	1 5 25	14	C– T3– T4– C– T3– T4↓ C– T3– T4–	Canli et al. [44]
N	*P. hypophthalmus*	Mn-NPs	91–95	4	C↑	Kumar et al. [112]
O	*C. carpio*	Pyrene	10–50 * 100 *	35	T3– T4– T3↓ T4↓	Shirdel et al. [46]
O	*C. gariepinus*	Bisphenol A	1.43 *	30	♀ FSH↓ LH↑ T↑ E↓	Hamed et al. [121]
O	*C. gariepinus*	4-nonylphenol	0.1 0.2 0.3	15	FSH– LH– T– E↑ FSH– LH– T↓ E↑ FSH↓ LH↑ T↓ E↑	Sayed et al. [122]
O	*D. rerio*	Tris(2-chloroethyl) phosphate	0.5 * 5 *	28	T3– T4↑ T3↓ T4↑	Tian et al. [123]
O	*D. rerio*	Tris(2-chloroethyl) phosphate	0.1 1.5	14 28 14 28	♀ E– T– ♂ E– T– ♀ E↓ T↓ ♂ E↓ T↓ ♀ E↓ T↓ ♂ E↓ T↓ ♀ E↓ T↓ ♂ E↓ T↓	Sutha et al. [124]
O	*D. rerio*	2-ethylhexyl diphenyl phosphate	2.5 * 50 * 250 *	21	♀ E– T↓ ♂ E– T– ♀ E– T↓ ♂ E↑ T↑ ♀ E↑ T– ♂ E↑ T↑	Yang et al. [125]
O	*D. rerio* embryos/larvae	KClO_4_	0.5–1 **** 1.5 ****	4	T3– T4– T3↑ T4↓	Di Paola et al. [108]
O	*D. rerio* embryos/larvae	N-(1,3-dimethylbutyl)-N′ -phenyl-p-phenylenediamine	2.2 * 22 * 220 *	5	GH– IGF↓ T3↓ T4– GH– IGF– T3↓ T4↑ GH↓ IGF↓ T3↓ T4↑	Peng et al. [126]
O	*D. rerio* embryos/larvae	Perfluorohexanoic acid	0.48 2.4 12	4	T3– T4↓ T3– T4↓ T3↑ T4↑	Zhang et al. [127]
O	*D. rerio* embryos/larvae	Tetrabromobisphenol A	50–100 * 200 *	5	T3– T4– T3↓ T4↑	Zhu et al. [128]
O	*D. rerio* embryos/larvae	Dichlorooctylisothiazolinone	0.003–0.3 * 3–30 *	4	T3– T4– T3↓ T4↓	Lee and Ji [129]
O	*D. rerio* embryos/larvae	Avobenzone	0.3–3 *** 10 *** 30 ***	5	T3– T4– T3– T4↓ T3↑ T4↓	Ka and Ji [130]
O	*D. rerio* embryos/larvae	Octinoxate	0.3–10 *** 30 ***	5	T3– T4– T3↓ T4↓	Ka and Ji [130]
O	*O. niloticus*	Ammonium chloride (NH_4_Cl)	3	4	♀ C↑ ♂ C↑	Zeitoun et al. [131]
O	*S. maximus*	Nitrate nitrogen (NO_3_-N)	50 200 400	60	T3↓ T4– GH– IGF– CRH– C– T3↓ T4– GH↓ IGF– CRH↑ C– T3↓ T4↓ GH↓ IGF↓ CRH↑ C↑	Yu et al. [132]
O	*T. rubripes*	Ammonia (NH_3_)	5	0.5–4	T3– T4– CRH– ACTH– C–	Gao et al. [133]
			50	0.5	T3– T4– CRH– ACTH– C↑	
				1–4	T3↑ T4↓ CRH↑ ACTH↑ C↑	
			100–150	0.5–4	T3↑ T4↓ CRH↑ ACTH↑ C↑	
Pd	*D. rerio* embryos	Pazopanib	10 ** 50 ** 100 **	4	T3– T4– TSH– T3– T4– TSH↑ T3↓ T4↓ TSH↑	Yang et al. [134]
Pd	*D. rerio* embryos	Axitinib	10 ** 50 ** 100 **	4	T3– T4– TSH↑ T3– T4– TSH↑ T3↓ T4↓ TSH↑	Yang et al. [134]
Pd	*D. rerio* embryos/larvae	Diclofenac	15.5–139.5 *	5	T3– T4↓	Wang et al. [135]
Pd	*D. rerio* embryos/larvae	Polyhexamethylene guanidine hydrochloride	4–40 * 400 *	7	T3– T4– T3↑ T4↓	Park et al. [136]
Pd	*D. rerio* embryos/larvae	Chlortetracycline hydrochloride	2	5	GH– FSH– LH– E–	Liu et al. [109]
Pd	*D. rerio* embryos/larvae	Oxytetracycline	2	5	GH– FSH– LH– E↑	Liu et al. [109]

* µg/L; ** nM; *** µM; **** mM. GCS—group of chemical substances; Em—metals and other elements; F—fungicides; H—herbicides; I—insecticides; M—microplastics; N—nanoparticles; O—other substances; Pd—pharmaceuticals and disinfectants. Exp. dur.—exposure duration; d—days; NPs—nanoparticles. *C. carpio—Cyprinus carpio, C. gariepinus—Clarias gariepinus, D. rerio—Danio rerio, H. molitrix—Hypophthalmichthys molitrix*, *L. catla*—*Labeo catla, L. rohita—Labeo rohita, M. salmoides—Micropterus salmoides, M. cephalus—Mugil cephalus, O. mykiss—Oncorhynchus mykiss, O. niloticus—Oreochromis niloticus, P. major—Pagrus major, P. hypophthalmus—Pangasianodon hypophthalmus, S. maximus—Scophthalmus maximus, S. schlegelii—Sebastes schlegelii, T. rubripes—Takifugu rubripes.* ACTH—adrenocorticotropic hormone; C—cortisol; CRH—corticotropin-releasing hormone; E—17β-estradiol or estrone; FSH—follicle-stimulating hormone; G—glucagon; GH—growth hormone; IGF—insulin-like growth factor; LH—luteinizing hormone; T—testosterone or 11-ketotestosterone; T3—triiodothyronine; T4—thyroxine; TSH—thyrotropin.

## 4. Conclusions

Our analysis of the studies in the current review showed that toxicity-induced changes in blood biochemical parameters in fish often depend on toxic agent concentration and exposure duration. Moreover, various parameters manifest different sensitivity to intoxication, and their changes may occur in different directions. Some biochemical parameters are biomarkers of general physiological stress (e.g., glucose, lactate, and ammonia). In contrast, others indicate dysfunctions of particular organs, including the liver (e.g., total protein, albumins, triglycerides, cholesterol, bilirubin, alanine aminotransferase, and aspartate aminotransferase) and kidney (creatinine), or suggest various metabolic disorders (lactate dehydrogenase). It seems that hormonal endpoints (such as measurements of thyroid hormones, sex steroids, cortisol, and insulin-like growth factors) are sensitive but nonspecific biomarkers of intoxication in fish. The interpretation of toxicological research results is additionally made more difficult due to the lack of generally accepted reference values of fish blood biochemical parameters. Thus, normal values should be obtained in each particular experiment or field study as values for fish not treated with tested toxicants or sampled from a non-polluted site of a reservoir. Despite the above-mentioned difficulties, we recommend using fish blood for biochemical analyses. However, in the case of early developmental stages of fish (embryos and larvae), when blood collection is impossible, the use of whole body homogenates for biochemical determinations is acceptable.

## Data Availability

The dataset supporting the reported results can be found in scientific databases, e.g., Web of Science or PubMed.

## Abbreviations

The following abbreviations are used in this manuscript:

ACTH	adrenocorticotropic hormone
Alb	albumins
ALP	alkaline phosphatase
ALT	alanine aminotransferase
AST	aspartate aminotransferase
C	cortisol
*C. carpio*	*Cyprinus carpio*
*C. gariepinus*	*Clarias gariepinus*
*C. idella*	*Ctenopharyngodon idella*
*C. mrigala*	*Cirrhinus mrigala*
*C. punctata*	*Channa punctata*
Cr	creatinine
CRH	Corticotropin-releasing hormone
d	days
*D. rerio*	*Danio rerio*
E	17β-estradiol or estrone
EDs	endocrine disruptors
Em	metals and other elements
Exp. dur.	exposure duration
F	fungicides
FSH	follicle-stimulating hormone
G	glucagon
GCS	group of chemical substance
GH	growth hormone
Glb	globulins
Glu	glucose
H	herbicides
*H. bidorsalis*	*Heterobranchus bidorsalis*
*H. molitrix*	*Hypophthalmichthys molitrix*
*H. nobilis*	*Hypophthalmichthys nobilis*
I	insecticides
IGF	insulin-like growth factor
*L. catla*	*Labeo catla*
*L. rohita*	*Labeo rohita*
Lct	lactate
LDH	lactate dehydrogenase
LH	luteinizing hormone
M	microplastics
*M. cephalus*	*Mugil cephalus*
*M. salmoides*	*Micropterus salmoides*
N	nanoparticles
n/a	not applicable
NH_3_	ammonia
NPs	nanoparticles
O	other substances
*O. mykiss*	*Oncorhynchus mykiss*
*O. niloticus*	*Oreochromis niloticus*
*P. fluviatilis*	*Perca fluviatilis*
*P. fulvidraco*	*Pelteobagrus fulvidraco*
*P. hypophthalmus*	*Pangasianodon hypophthalmus*
*P. major*	*Pagrus major*
Pd	pharmaceuticals and disinfectants
*R. quelen*	*Rhamdia quelen*
*S. maximus*	*Scophthalmus maximus*
*S. schlegelii*	*Sebastes schlegelii*
T	testosterone or 11-ketotestosterone
*T. rubripes*	*Takifugu rubripes*
T3	triiodothyronine
T4	thyroxine
TB	total bilirubin
TC	total cholesterol
Tg	triglycerides
TP	total protein
TSH	thyrotropin
UAc	uric acid
Ur	urea
UrN	urea nitrogen
*V. vimba*	*Vimba vimba*
